# Hyaluronan Oligosaccharides Induce MMP-1 and -3 via Transcriptional Activation of NF-κB and p38 MAPK in Rheumatoid Synovial Fibroblasts

**DOI:** 10.1371/journal.pone.0161875

**Published:** 2016-08-26

**Authors:** Masahiro Hanabayashi, Nobunori Takahashi, Yasumori Sobue, Shinya Hirabara, Naoki Ishiguro, Toshihisa Kojima

**Affiliations:** Department of Orthopedic Surgery and Rheumatology, Nagoya University Graduate School of Medicine, Nagoya, Japan; University of Patras, GREECE

## Abstract

**Objective:**

To explore the effect of hyaluronan oligosaccharides (HAoligos) on interactions between HA and its principal receptor, CD44, in rheumatoid synovial fibroblasts (RSFs) and matrix metalloproteinase (MMP) production.

**Methods:**

RSFs were isolated from rheumatoid synovial tissue. HA distribution was visualized by immunocytochemistry. MMP-1 and MMP-3 induction was analyzed by real-time RT-PCR and immunoblotting. The interaction between HAoligos and their MMP-producing receptors was tested by blocking with anti-CD44 and anti-Toll-like receptor 4 (TLR-4). Phosphorylation of nuclear factor κB (NF-κB) and mitogen-activated protein kinase (MAPK) was analyzed by immunoblotting.

**Results:**

Endogenous HA decreased after treatment with HAoligos, while MMP-1 and MMP-3 expression increased in a dose-dependent manner. Pretreatment with anti-CD44 or anti-TLR-4 antibody significantly reduced the effect of HAoligos on MMP-1 and MMP-3 mRNA expression. NF-κB and p38 MAPK phosphorylation was enhanced by HAoligos pretreated with anti-TLR-4, and HAoligo-induced MMP production was blocked with an inhibitor of NF-κB and p38 MAPK pathways.

**Conclusions:**

Disruptive changes in CD44-HA interactions by HAoligos enhanced MMP-1 and MMP-3 production via activation of NF-κB and p38 MAPK signaling pathways in RSFs.

## Introduction

Rheumatoid arthritis (RA) is a systemic inflammatory disease characterized by joint destruction induced by hyperplasia and chronic inflammation of synovial membranes. Activated fibroblast-like synoviocytes in the lining layer of the synovium contribute significantly to cartilage degradation [1, 2]. Rheumatoid synovial fibroblasts (RSFs) in particular up-regulate the expression of matrix metalloproteinases (MMPs), which are key enzymes that degrade cartilaginous and bone matrices [3].

MMP-1 and MMP-3 are the main MMPs produced by fibroblasts and macrophage-like cells in the synovium, with significantly higher levels found in the synovial fluid of patients with RA compared to patients with osteoarthritis [4, 5]. These MMPs not only degrade collagens, proteoglycans, and other extracellular matrix (ECM) macromolecules in cartilage, but also activate other MMPs [6]. The actions of MMP-1 and MMP-3 lead to destruction of articular cartilage and subchondral bone, resulting in joint deformity and severe pain for patients with RA. It is therefore vital to elucidate the mechanisms of MMP-induced joint destruction and develop targeted treatment plans.

Many connective tissue cells have a large hyaluronan (HA) and proteoglycan-rich pericellular matrix that is tethered to the cell surface via interactions with primary HA receptor CD44 [7–9]. HA is a high-molecular weight polysaccharide consisting of repeating disaccharide glucuronic acid and *N*-acetylglucosamine units [10]; it is also an abundant component of ECMs and plays a role in regulating cell behavior during embryonic development, healing processes, inflammation, and tumor development [11].

A previous study found that decreasing extracellular HA in chondrocytes induced stimulation of catabolic genes involved in matrix degradation, such as MMPs [12]. However, the effects in RSFs are still uncertain, despite RSFs being major regulators of local inflammation in RA joints. In one study, the concentration of HA in synovial fluid of patients with RA was significantly less compared to healthy individuals [13]. In other studies, lower concentrations of HA were found in the synovia of patients with RA [14, 15]. These observations raise the possibility that decreased pericellular HA levels contribute to MMP expression in RSFs.

HA oligosaccharides (HAoligos), of the size of a HA hexasaccharide—decasaccharide, can compete with the binding of high-molecular weight HA (HMW-HA) to CD44 to displace it and the proteoglycan-rich pericellular matrix from the cell surface [7, 8, 12, 16–22]. Loss of the cell-associated pericellular matrix and uncoupling of HA from CD44 results in signal transduction events with outcomes based on cell type [8, 12, 16, 18–21]. The current CD44-HA model clusters CD44 receptors in the plasma membrane using multivalent interactions with HMW-HA; the cluster is reversed with the addition of excess HAoligos that displace endogenous HA and generate monovalent interactions between itself and CD44 [11, 16, 21]. CD44 is a transmembrane receptor with a short intracellular tail domain but no inherent kinase activity [23]; signaling events induced by the unclustering of CD44 by HAoligos are thus likely to involve the activation of CD44-associated proteins, such as kinases and cytoskeletal elements [24]. Although *in vivo* fragmentation of HA has yet to be elucidated, activated fibroblasts secrete hyaluronidase to degrade HMW-HA into fragments during inflammation [25, 26]. This degradation can be augmented by reactive oxygen species produced at inflammation sites [27, 28].

In this study, HAoligos were used as a molecular tool to mimic decreasing HA and disrupted CD44-HA interactions. We investigated the effect of HAoligo treatment on MMP production in RSFs and explored the CD44-dependent signaling pathway responsible for MMP production by HAoligos.

## Materials and Methods

### Reagents

Mouse anti-human CD44 (clone BU52) monoclonal antibody was purchased from Ancell (Bayport, MN, USA). Mouse anti-human Toll-like receptor 4 (TLR-4; clone HTA125) monoclonal antibody was purchased from Abcam (Cambridge, UK). For immunoblot analysis, mouse anti-human MMP-1 (clone 41-1E5) and MMP-3 (clone 55-2A4) monoclonal antibodies were purchased from Daiichi Fine Chemical (Toyama, Japan). Rabbit anti-human nuclear factor κB (NF-κB) p65 (#3987), phospho-NF-κB p65 (#3033), p38 mitogen-activated protein (p38 MAPK; #9212), phospho-p38 MAPK (#4511), Erk1/2 (#4695), phospho-Erk1/2 (#4370), c-Jun N-terminal kinase (JNK; #9252), phospho-JNK (#9251), and β-actin antibodies (#4970) were purchased from Cell Signaling Technology (Beverly, MA, USA). Horseradish peroxidase (HRP)-conjugated goat anti-mouse (#7076) and anti-rabbit (#7074) IgG were also obtained from Cell Signaling Technology. Dulbecco’s modified Eagle’s medium (DMEM) and trypsin ethylenediaminetetraacetic acid (EDTA) were obtained from Sigma-Aldrich Co. (St. Louis, MO, USA). Fetal bovine serum (FBS) was purchased from PAA Laboratories GmbH (Pasching, Austria).

### Cell culture

Synovial tissues were obtained from six patients with RA at the site of total knee replacement surgery that fulfilled the revised criteria of the American College of Rheumatology (1987)[29]. Written informed consent was obtained from each patient prior to the study, and experiments were performed in accordance with a study protocol approved by the Ethics committee of Nagoya University. The tissue was minced into small pieces and digested with 4 mg/ml collagenase (Sigma-Aldrich) in DMEM for two hours at 37°C. After removing tissue debris by filtering through a 70-μm cell strainer (BD Biosciences, Franklin Lakes, NJ, USA), cells were centrifuged for five minutes at 1,500 rpm. The cell pellet was resuspended in DMEM containing 100 U/ml penicillin, 100 μg/ml streptomycin, 0.25 μg/ml amphotericin B (Invitrogen, Carlsbad, CA, USA), and 10% heat-inactivated FBS, plated on 75 cm^2^ tissue culture flasks (TPP Techno Plastic Products AG, Schaffhausen, Switzerland), and cultured at 37°C in a humidified 5% CO_2_ atmosphere. At confluence, cells were passaged 1:3 by treatment with EDTA. Cells with 3–5 passages were considered to be RSFs. Before experiments, RSFs were seeded into six-well culture plates (TPP) at a density of 4.0 × 10^5^ cells/well. In some experiments, RSFs were seeded into 22.1 cm^2^ dishes (TPP) at a density of 1.0 × 10^6^ cells/dish.

### Preparation of hyaluronan oligosaccharides

HAoligos were refined as previously described [30]. Briefly, HMW-HA from human umbilical cord (Sigma-Aldrich) was dissolved in 0.1 M sodium acetate buffer (pH 5.0) containing 0.15 M NaCl and treated with bovine testicular hyaluronidase (320 units/mg, type I-S; Sigma-Aldrich) for 16 hours at 37°C. Duration of incubation and temperature were selected to best generate a predominant proportion of HA hexasaccharides [20]. At the end of the reaction, hyaluronidase was heat-inactivated, precipitated with 10% trichloroacetic acid, and pelleted by centrifugation at 2,000 rpm for 10 minutes. The oligosaccharide-containing supernatant was dialyzed in 1,000 molecular weight cut-off (MWCO) dialysis tubing (Spectrum Medical Industries, Houston, TX, USA) with four changes of H_2_O over 48 hours, lyophilized, and redissolved in sterile phosphate buffer saline (PBS).

### Hyaluronan staining with hyaluronic acid binding protein

HA distribution in RSFs after treatment with HAoligos was visualized by staining with a biotinylated HA binding protein (b-HABP; Seikagaku Biobusiness Co., Tokyo, Japan) that has a high affinity for the decasaccharide unit of HA. RSFs were seeded into chamber slides (BD Biosciences) for 48 hours and treated with or without 250 μg/ml HAoligos for one or three hours. Cells were then fixed with 4% paraformaldehyde, buffered with PBS at room temperature for one hour, treated with 0.3% H_2_O_2_ in PBS for 30 minutes at room temperature to block internal peroxidase activity, and incubated with 1% bovine serum albumin in PBS for one hour at room temperature. Cells were incubated with 2.0 μg/ml b-HABP for one hour at room temperature, and bound b-HABP was detected by the addition of streptavidin-peroxidase reagents and diaminobenzidine (DAB)-containing substrate solution (Nichirei Biosciences Inc., Tokyo, Japan). As a negative control, cells were pretreated with 100 units/ml bovine testicular hyaluronidase (Sigma-Aldrich) for three hours before incubation with b-HABP.

### Hyaluronan oligosaccharide treatment

Forty-eight hours after seeding, RSFs were washed with PBS 2 times and brought to serum-free conditions overnight prior to beginning the experiment. To investigate MMP-1 and MMP-3 mRNA expression and protein secretion, RSFs were treated with various concentrations of HAoligos (0–500 μg/ml) in serum-free DMEM for 12 or 24 hours. In some experiments, cells were pretreated with 5 μg/ml anti-CD44 or 5 μg/ml anti-TLR-4 antibody for one hour before HAoligo treatment. To investigate intracellular signaling pathways, RSFs were pretreated with anti-TLR-4 antibody followed by 250 μg/ml HAoligos for 0–120 min. For signal inhibition assays, RSFs were pretreated with inhibitors for NF-κB (Helenalin; Merck KGaA, Darmstadt, Germany) or p38 MAPK (SB203580; Merck KGaA) for one hour, followed by the addition of 250 μg/ml HAoligos for 24 hours in the presence or absence of each inhibitor. To examine the effects of HMW-HA on HAoligo-induced MMP mRNA expression, RSFs were pretreated with 250 μg/ml HAoligos for 12 hours and followed with 250 μg/ml HAoligos or 1 mg/ml HMW-HA (600–1,200 kDa; Seikagaku Co., Tokyo, Japan) for 12 hours.

### Real-time RT-PCR

Total RNA was isolated from RSF cultures using the illustra triplePrep kit (GE Healthcare UK Ltd, Buckinghamshire, UK), and reverse transcription was performed with the High Capacity cDNA Reverse Transcription Kit (Applied Biosystems, CA, USA) according to manufacturer protocols. Equal amounts of each reverse transcription product were amplified by real-time RT-PCR using the LightCycler FastStart DNA Master SYBR Green I kit (Roche, Mannheim, Germany). Thermal cycling and fluorescence detection were performed using the LightCycler 480 System (Roche, USA). MMP-1 and MMP-3 mRNA expression levels were normalized to glyceraldehyde phosphate dehydrogenase (GAPDH) as fold change compared to control at each time point. MMP-1, MMP-3, and GAPDH primer pairs are as follows:

MMP-1, forward 5’-CTGGGAGCAAACACATCTGA-3’,

reverse 5’-CTGGTTGAAAAGCATGAGCA-3’; Gen Bank ID: NM_002421.

MMP-3, forward 5’-GAAGACTTTCCAGGGATTGACT-3’,

reverse 5’-GTGCCTTCTACTACTCTTTCAAC -3’; Gen Bank ID: NM_002422.

GAPDH, forward 5’-TGAACGGGAAGCTCACTGG-3’,

reverse 5’-TCCACCACCCTGTTGCTGTA-3’; Gen Bank ID: NM_008084.

All primers were designed by Nihon Gene Research Laboratories (Miyagi, Japan).

### DNA assay

After conditioned media was collected, genomic DNA was isolated from RSF cultures using the illustra triplePrep kit (GE Healthcare). DNA content was determined by BioPhotometer plus (Eppendorf, Hamburg, Germany).

### Western blot analysis

To investigate MMP-1 and MMP-3 secreted into conditioned media, medium samples were collected in the presence of a protease inhibitor cocktail (Thermo Fisher Scientific Inc., Waltham, MA, USA), according to manufacturer protocols. Samples were centrifuged at 14,000 g for 10 minutes at 4°C. After centrifugation, supernatants were mixed with Lane Marker Reducing Sample buffer (Thermo Fisher Scientific) and heated at 80°C for 10 minutes. Samples were then separated by electrophoresis on 10% SDS-polyacrylamide gel and electroblotting onto polyvinylidene fluoride membranes using a Mini Trans-Blot apparatus (BIO-RAD, Hercules, CA, USA). The sample amount loaded was determined by the DNA content of RSFs in each well. After blocking in 0.5% skim milk for one hour, membranes were incubated overnight at 4°C with primary antibodies against MMP-1 or MMP-3. After washing with PBS containing 0.1% Tween 20, membranes were incubated for 30 minutes with HRP–conjugated secondary antibodies. Detection was performed using chemiluminescence with West Pico Chemiluminescent Substrate (Thermo Fisher Scientific).

To investigate intracellular signaling pathways, total protein was extracted from RSFs treated 0–120 minutes with or without HAoligos using Cell Lysis Buffer (Cell Signaling Technology) containing protease and phosphatase inhibitor cocktails (Thermo Fisher Scientific). Protein content was determined using protein assay reagent (BIO-RAD) and BSA as a standard. In some cases, blots were treated with striping buffer and re-probed using another primary antibody. Band intensities were captured with a digital image scanner and quantified using densitometry software (CS Analyzer 3.0; ATTO, Tokyo, Japan).

### Casein zymography

To investigate the activity of MMPs secreted into conditioned media, media samples were collected, centrifuged at 14,000 g for 10 minutes at 4°C, and the resultant supernatants were incubated at 37°C with 2.5 mM p-aminophenylmercuric acetate for four hours to activate MMPs. Samples were mixed with Tris-Glycine SDS Sample Buffer (Thermo Fisher Scientific) and separated by electrophoresis on 12% Zymogram (Casein) Protein Gels (Thermo Fisher Scientific). The amount of sample loaded was determined based on the DNA content of RSFs in each well. After electrophoresis, gels were incubated in Zymogram Renaturing Buffer (Thermo Fisher Scientific) for 30 minutes at room temperature with gentle agitation and subsequently in Zymogram Developing Buffer (Thermo Fisher Scientific) for 30 minutes. Gels were then incubated in fresh Zymogram Developing Buffer at 37°C overnight, stained with Coomassie brilliant blue overnight, and bleached with 45% methanol / 10% acetic acid.

### Statistical analysis

Values are expressed as mean ± SD. Statistical significance was analyzed by Student’s t-test. P values less than 0.05 were considered significant. All analyses were conducted with SPSS for Windows ver. 19 (SPSS, Chicago, IL).

## Results

### HAoligo treatment of RSFs reduces endogenous HA

**Fig 1:**
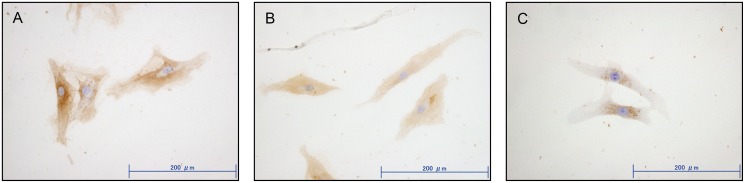
Effect of hyaluronan oligosaccharides (HAoligos) on endogenous HA deposition in rheumatoid synovial fibroblasts (RSFs). HA accumulation in RSFs after treatment with or without HAoligos was visualized by diaminobenzidine (DAB) staining using a biotinylated HA binding protein. **A**, Control medium. **B**, 250 mg/ml HAoligos. **C**, 400 units/ml bovine testicular hyaluronidase. Original magnification × 400, scale bar: 200 μm.

To assess whether HAoligos can displace HMW-HA from the cell surface, RSFs were cultured with or without HAoligos for three hours. DAB staining for HA (brown) was abundant on the cell surface or in the cytoplasm of untreated RSFs (Fig 1A). In contrast, stainable HA decreased after treatment with HAoligos (Fig 1B) or hyaluronidase (Fig 1C). After a one hour incubation with HAoligos, slight displacement of HMW-HA was observed (S1 Fig). These results suggest that HAoligos can displace HMW-HA from the cell surface.

### Effect of HAoligos on MMP-1 and MMP-3 mRNA expression and protein secretion

**Fig 2:**
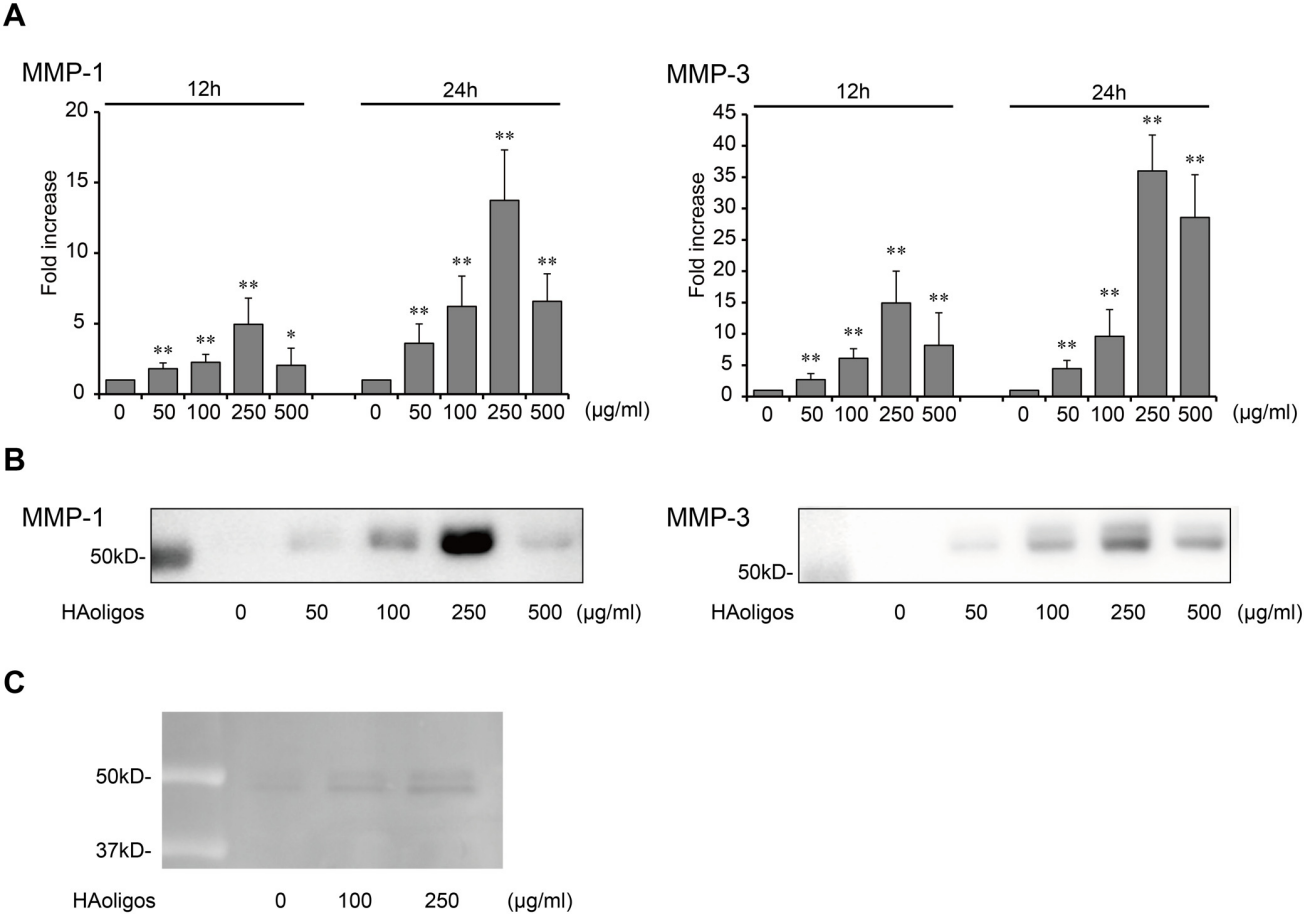
Expression levels of matrix metalloproteinase (MMP) mRNA and protein secretion induced by HAoligo treatment. RSFs were treated with various concentrations of HAoligos (0–500 μg/ml). **A**, MMP-1 and MMP-3 mRNA expression levels after 12 or 24 hours of HAoligo treatment as detected by quantitative real-time RT-PCR. Results shown are fold change in mRNA copy number. Values are mean ± S.D. of three independent experiments analyzed in triplicate, and are expressed as n-fold increase compared to untreated control. *p<0.05 and **p<0.01 vs. control. **B**, MMP-1 and MMP-3 protein secretion in conditioned media after 24 hours of HAoligo treatment as evaluated by immunoblot analysis. The amount of sample separated by SDS-PAGE before transfer and immunostaining was determined by the DNA content of RSFs. Results shown are representative of three experiments with similar results. C, The activity of MMPs secreted into conditioned media was evaluated by casein zymography. RSFs were stimulated with 0, 100, or 250 μg/ml HAoligos for 24 hours. The image was digitally inverted to generate dark bands on a light background.

To explore the mechanism of MMP-1 and MMP-3 induction, cultured RSFs were treated with various concentrations of HAoligos for 12 or 24 hours. As shown in Fig 2A, induction of MMP-1 mRNA by HAoligos was first observed at 50 μg/ml and increased in a dose-dependent manner, with maximum enhancement at 250 μg/ml. Consistent with the mRNA findings, MMP-1 protein secreted into culture media also increased with HAoligo treatment (Fig 2B). MMP-3 mRNA expression and protein secretion were similar to that observed with MMP-1. Moreover, MMPs secreted into conditioned media had caseinolytic activity (Fig 2C). These results indicate that HAoligos affect MMP-1 and MMP-3 induction at both the transcript and protein level.

### Involvement of TLR-4 and CD44 in HAoligo-induced MMP mRNA expression

**Fig 3:**
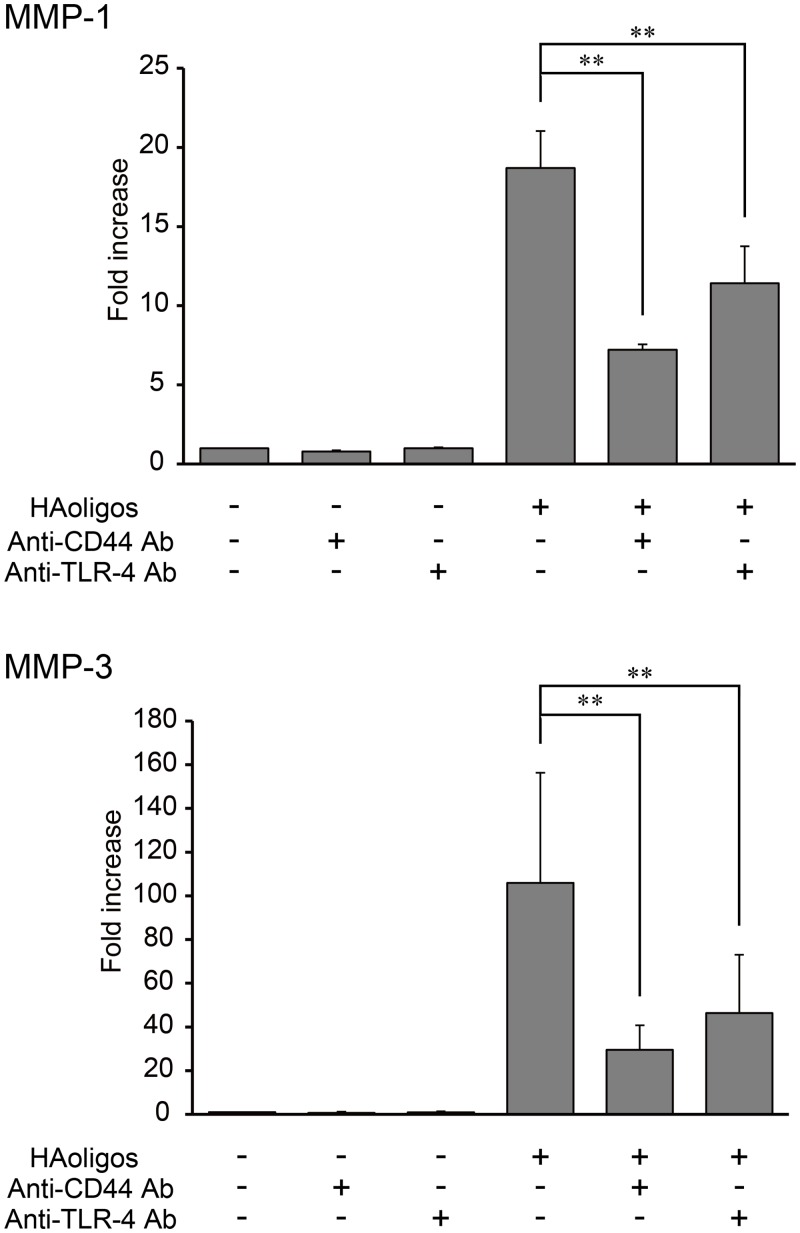
Expression levels of MMP mRNA induced by HAoligo treatment with or without CD44 and Toll-like receptor 4 (TLR-4) neutralizing antibodies. RSFs were pre-treated for one hour with or without 5μg/ml anti-CD44 and 5μg/ml TLR-4 antibodies and followed by HAoligo treatment (250 μg/ml) for 24 hours. MMP-1 and MMP-3 expression levels were determined by quantitative real-time RT-PCR. Values are mean ± S.D of three independent experiments analyzed in triplicate, and are expressed as n-fold increase compared to untreated control. **p<0.01 vs. control.

Pattern recognition receptors such as TLR-4 are involved in HAoligo-induced cell signaling [31–36], raising the possibility that they play a role in HAoligo-mediated MMP production in RSFs. The effect of HAoligos on MMP mRNA expression via interaction with CD44 and TLR-4 was thus evaluated using neutralizing antibodies against each receptor. As shown in Fig 3, MMP-1 and MMP-3 mRNA expression induced by 250 μg/ml HAoligo treatment significantly decreased with a 5 μg/ml CD44 neutralizing antibody. Similar results were obtained in RSFs pretreated with a 5 μg/ml TLR-4 neutralizing antibody. Furthermore, no cell toxicity was observed for the indicated concentrations of antibodies (data not shown). These findings show that HAoligos interact with CD44 as well as TLR-4 to induce MMP mRNA expression.

### TLR-4-independent activation of NF-κB and p38 MAPK by HAoligos

**Fig 4:**
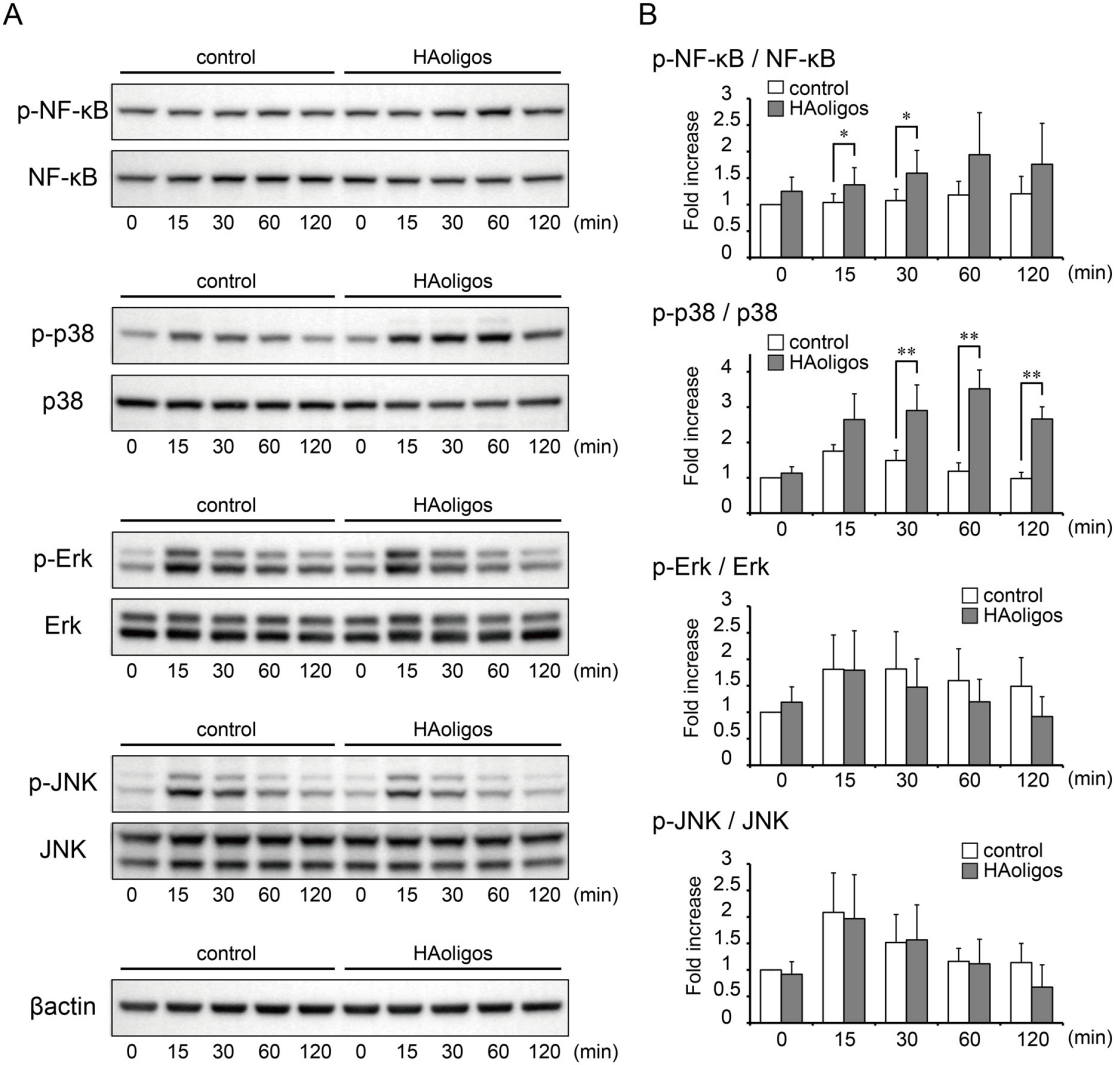
Intracellular signaling cascades stimulated by HAoligos with pre- and co-incubation with a TLR4 neutralizing antibody. RSFs were pre-treated for one hour with an anti-TLR4 antibody, followed by HAoligo treatment (250 μg/ml). Phosphate buffered saline was substituted as a control. **A**, Phospho-nuclear factor κB (NF-κB), NF-κB, phospho-p38 mitogen-activated protein kinase (MAPK), p38 MAPK, phospho-Erk, Erk, phospho-c-Jun N-terminal kinase (JNK), and JNK, as evaluated by immunoblot analysis of RSFs derived from three patients with RA. Results shown are representative of three experiments with similar results. **B**, Densitometry of phospho-NF-κB/NF-κB, phospho-p38 MAPK/p38 MAPK, phospho-ERK/ERK and phospho-JNK/JNK. Values are mean ± S.D. of three independent experiments and expressed as n-fold increase compared to untreated control at time 0. *p<0.05 and **p<0.01 vs. control.

Activation of NF-κB, p38 MAPK, ERK, and JNK by HAoligo treatment was examined in RSFs. After stimulation with 250 μg/ml HAoligos, the cell lysate was analyzed by immunoblot. HAoligos enhanced the phosphorylation of NF-κB and p38 MAPK, while JNK and Erk phosphorylation levels were equivalent to control samples (S5 Fig). To assess the involvement of TLR-4 pathways, RSFs were also pretreated with a TLR4 neutralizing antibody. HAoligo treatment of these cells increased the phosphorylation of NF-κB and p38 MAPK, while ERK and JNK phosphorylation levels were equivalent to control samples (Fig 4A and 4B). Total protein levels were unchanged during the experiment. These results indicate that HAoligo treatment resulted in activation of NF-κB and p38 MAPK through a TLR-4 independent pathway.

### Requirement of NF-κB and p38 MAPK for MMP production in HAoligo-stimulated RSFs

**Fig 5:**
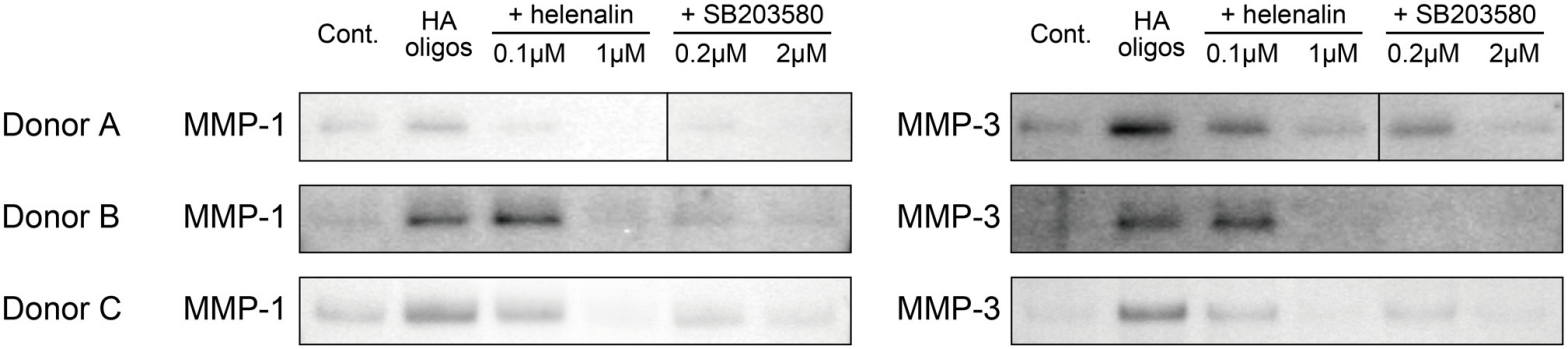
Effects of chemical inhibitors on MMP production induced by HAoligo treatment. After pretreatment with helenalin or SB203580 for one hour at the indicated concentrations, RSFs were stimulated with 250 μg/ml HAoligos for 24 hours in the presence or absence of each inhibitor. The conditioned media was analyzed by immunoblot using antibodies against MMP-1 and MMP-3. The amount of sample separated by SDS-PAGE before transfer and immunostaining was determined by the DNA content of RSFs in each well. RSFs derived from three patients with RA were used. In donor A, SB203580 lanes were not adjacent to helenalin lanes. Thus, for clarity, SB203580 lanes were cut out and displayed next to helenalin lanes.

To investigate whether NF-κB and p38 MAPK were responsible for the production of MMP-1 and MMP-3 stimulated by HAoligos in RSFs, selective protein kinase inhibitors were used. As shown in Fig 5, pre- and co-incubation with inhibitors for NF-κB (helenalin) or p38 MAPK (SB203580) resulted in significant decreases in MMP-1 and MMP-3 production stimulated by HAoligos. These findings suggest that MMP-1 and MMP-3 production induced by HAoligos involves NF-κB and p38 MAPK.

### Effect of HMW-HA on MMP-1 and MMP-3 mRNA expression induced by HAoligo treatment

**Fig 6:**
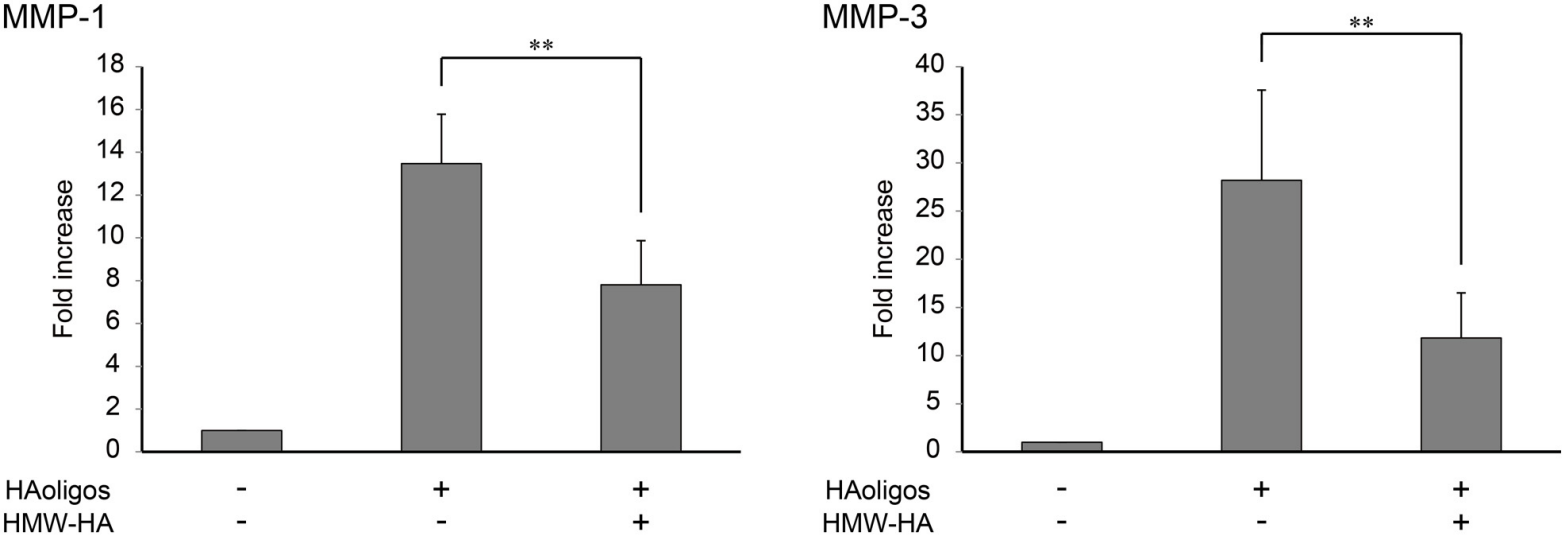
Effect of HMW-HA on MMP mRNA expression induced by HAoligo treatment. RSFs were treated with HAoligos (250 μg/ml) for 12 hours. In next 12 hours, RSFs were treated with HAoligos (250 μg/ml) or HMW-HA (1mg/ml). MMP-1 and MMP-3 expression levels were determined by quantitative real-time RT-PCR. Values are presented as mean ± S.D of two independent experiments analyzed in triplicate, and are expressed as n-fold increase relative to untreated control. **p<0.01 vs. HAoligos.

To determine whether HMW-HA added to cells pretreated with HAoligos could reduce MMP-1and MMP-3 up-regulation, MMP mRNA expression was analyzed. As shown in Fig 6, HAoligo-induced MMP-1 and MMP-3 mRNA expression was significantly attenuated following the addition of HMW-HA. These results suggest that HMW-HA reduces MMP-1 and MMP-3 expression induced by HAoligos

## Discussion

In the present study, low-molecular mass HAoligos were used to disrupt CD44-HA interactions. We demonstrated that these small oligosaccharides displace HMW-HA from the cell surface (Fig 1) and influence the up-regulation of MMP-1 and MMP-3 at both the transcript and protein level, thereby leading to matrix degradation (Fig 2). Previous studies demonstrated that HAoligos increased MMPs in articular chondrocytes [12, 30, 37]; however, fibroblast-like synoviocytes are more critical for understanding the pathology of RA joint destruction rather than chondrocytes in cartilage tissue. Synoviocytes are the major cellular constituent of synovium, the principal tissue involved in the regulation of inflamed joints, and the primary reason HAoligo treatment in RSFs was explored.

The receptor involved in HA-mediated MMP induction remains somewhat ambiguous, as HA can associate with several cell surface molecules. Although CD44 is the best known HA receptor [9], HAoligos can also interact with TLR-4 to promote cytokine and MMP up-regulation [31–36]. TLRs are pattern recognition receptors found in a variety of cells and tissues; they can detect invading microbes as well as sense tissue damage and, in response, induce expression of pro-inflammatory genes [38, 39]. To determine the HA receptor involved in MMP up-regulation, we performed an inhibition assay using neutralizing antibodies to CD44 and TLR-4. Our data showed that although TLR-4 is involved in HAoligo-induced MMP-1 and MMP-3 mRNA expression in RSFs, CD44 mediates HAoligo-initiated cell signaling (Fig 3).

NF-κB and MAPK families play a critical role in the induction of MMPs [40]. Previous studies with chondrocytes reported that NF-κB and p38 MAPK are involved in MMP-3 and MMP-13 production through CD44-HAoligos interactions [12, 30]. Another study determined that p38 MAPK plays a role in signal transduction for MMP-1 induced by HAoligos in periodontal ligament cells [41]. We found that CD44-HAoligo interaction increased the activation of NF-κB and p38 MAPK, which led to MMP-1 and MMP-3 secretion in RSFs (Figs 4 and 5). A possible explanation for the different signaling pathways responsible for MMP production may be that it is dependent on cell type.

Recent clinical trials have clearly shown that biologics targeting inflammatory cytokines can achieve good clinical results in the treatment of RA patients; however, complete suppression of joint destruction has yet to be achieved. Joint destruction pathways other than cytokines are believed to contribute to RA pathogenesis independent of biologic treatment strategies. Innate immunity is one possible pathway, and our findings suggest that decreasing HA in RA joints may also lead to destroyed joints.

HMW-HA is a biodegradable, biocompatible, nontoxic, non-immunogenic, and non-inflammatory linear polysaccharide. Given its natural presence in the body, HA has advantages over synthesized molecules in terms of biophysiological effects and possible side effects that make it an attractive drug. Recent studies have shown that HA plays an important role in joint lubrication, protects articular cartilage from damage, and acts as a biological inhibitor of inflammation and degradation of joints by down-regulating gene expression of osteoarthritis-associated enzymes, such as MMP-3 in fibroblast-like synoviocytes [42], and inhibiting MMP-1 and MMP-3 production by RSFs [43, 44]. Restoration of HA to normal levels in RA synovial fluid could therefore decrease elevated MMP-1 and MMP-3 levels in RA joints. Although some studies have reported the clinical efficacy of HA intra-articular injection in RA patients [45, 46], only a few have reported that HA has the potential to prevent joint destruction. In the present study, HMW-HA attenuated the HAoligo-induced upregulation of MMP-1 and MMP-3 mRNA (Fig 6). Our findings provide new data to support the efficacy of HA treatment in RA.

There are some limitations to this study. First, the prepared HAoligos may have been contaminated, e.g., by hyaluronidase. However, we performed preliminary experiments to rule out contamination. For instance, we found that the HA-free solution did not affect the expression levels of MMP-1 and -3, suggesting that the HA-free solution was free of contamination and contained only HMW-HA (S2 Fig). Furthermore, HMW-HA was able to rescue mRNA levels of MMP-1 and -3 induced by HAoligos. These findings collectively suggest that the HMW-HA used was essentially free of contamination. Second, it is possible that other untested HA receptors, such as the receptor for hyaluronan-mediated motility (RHAMM), may have caused the observed effects. Further studies will be needed to determine whether signaling through other receptors are affected by treatment with HAoligos. Third, other effects of HAoligos on RSFs were not analyzed. This study demonstrated that HAoligos increased the expression of matrix degradation-related factors in RSFs. In other cell types, such as articular chondrocytes, HAoligos reportedly induce both catabolic and anabolic factors simultaneously [30]. Thus, further studies regarding other effects of HAoligos in RSFs are warranted.

In conclusion, we confirmed that disruptive changes in CD44-HA interactions by HAoligos enhance MMP-1 and MMP-3 production via activation of NF-κB and p38 MAPK signaling pathways in RSFs. This study illustrates that loss of the pericellular matrix leads to the activation of a catabolic cascade by RSFs, and the joint destruction pathway observed could provide new evidence for the biological effects of intra-articular supplementation of HMW-HA in inflammatory RA joints.

## Supporting Information

S1 Fig HA distribution in rheumatoid synovial fibroblasts (RSFs) after treatment with hyaluronan oligosaccharides (HAoligos). HA accumulation in RSFs after treatment with or without HAoligos for one hour was visualized by diaminobenzidine (DAB) staining using a biotinylated HA binding protein. Compared to results of longer incubation (three hours), slighter displacement of HMW-HA was observed after incubation with HAoligos for one hour. **A**, Control medium. **B**, 250 mg/ml HAoligos. Original magnification × 400, scale bar: 200 μm.
(TIF)

S2 Fig Effect of hyaluronan-free solution on MMP mRNA expression. Hyaluronan (HA)-free solution was prepared in the same manner as the preparation of hyaluronan oligosaccharides, but without HMW-HA, and dialyzed with PBS in the final dialysis. HAoligos in PBS were also prepared using conventional methods and dialyzed with PBS in the final dialysis. The concentration of HAoligos in PBS was calculated using the weight after lyophilization. RSFs were treated with the same amount of HA-free solution as the amount of HAoligos in PBS. HAoligos in PBS significantly affected MMP mRNA expression, whereas the HA-free solution did not. *p<0.05 and **p<0.01 vs. HAoligos in PBS.
(TIF)

S3 Fig Effects of isotype-matched control IgG on MMP mRNA expression induced by HAoligos. RSFs were pre-treated for one hour with or without antibodies (5 μg/ml), and followed by treatment with HAoligos (250 μg/ml) for 24 hours. MMP mRNA expression induced by HAoligos significantly decreased when RSFs were pre-treated with CD44 or TLR-4 neutralizing antibodies. In contrast, isotype-matched control IgG of anti-CD44 or -TLR4 antibodies did not reduce HAoligo-induced MMP mRNA expression. IgG1: isotype-matched control IgG of anti-CD44 antibody (Ancell). IgG2a: isotype-matched control IgG of anti-CD44 TLR4 antibody (Abcam). *p<0.05 and **p<0.01 vs. control IgG.
(TIF)

S4 Fig Suppressive effect of BU52 and BU75 anti-CD44 antibodies on MMP mRNA expression induced by HAoligos. RSFs were pre-treated for one hour with or without antibodies (5 μg/ml), and followed by treatment with HAoligos (250 μg/ml) for 24 hours. The suppressive effect of the BU52 antibody on MMP mRNA expression induced by HAoligos was comparable to that of the BU75 antibody (Ancell). **p<0.01 vs HAoligos.
(TIF)

S5 Fig Intracellular signaling pathways stimulated by HAoligos. Total protein was extracted from RSFs treated for 0–120 minutes with or without HAoligos (250 μg/ml). Phosphate buffered saline was used as the control. Levels of phospho-NF-κB, NF-κB, phospho-p38 MAPK, p38 MAPK, phospho-Erk, Erk, phospho- JNK, and JNK were evaluated by immunoblot analysis. HAoligos enhanced the phosphorylation of NF-κB and p38 MAPK, while JNK and Erk phosphorylation levels were equivalent to control samples.
(TIF)
