# Supplementary figures and images for: Hyaluronan Oligosaccharides Induce MMP-1 and -3 via Transcriptional Activation of NF-κB and p38 MAPK in Rheumatoid Synovial Fibroblasts
Source: PLoS One. 2016 Aug 26;11(8):e0161875. doi: 10.1371/journal.pone.0161875 (PMC5001728; doi:10.1371/journal.pone.0161875)

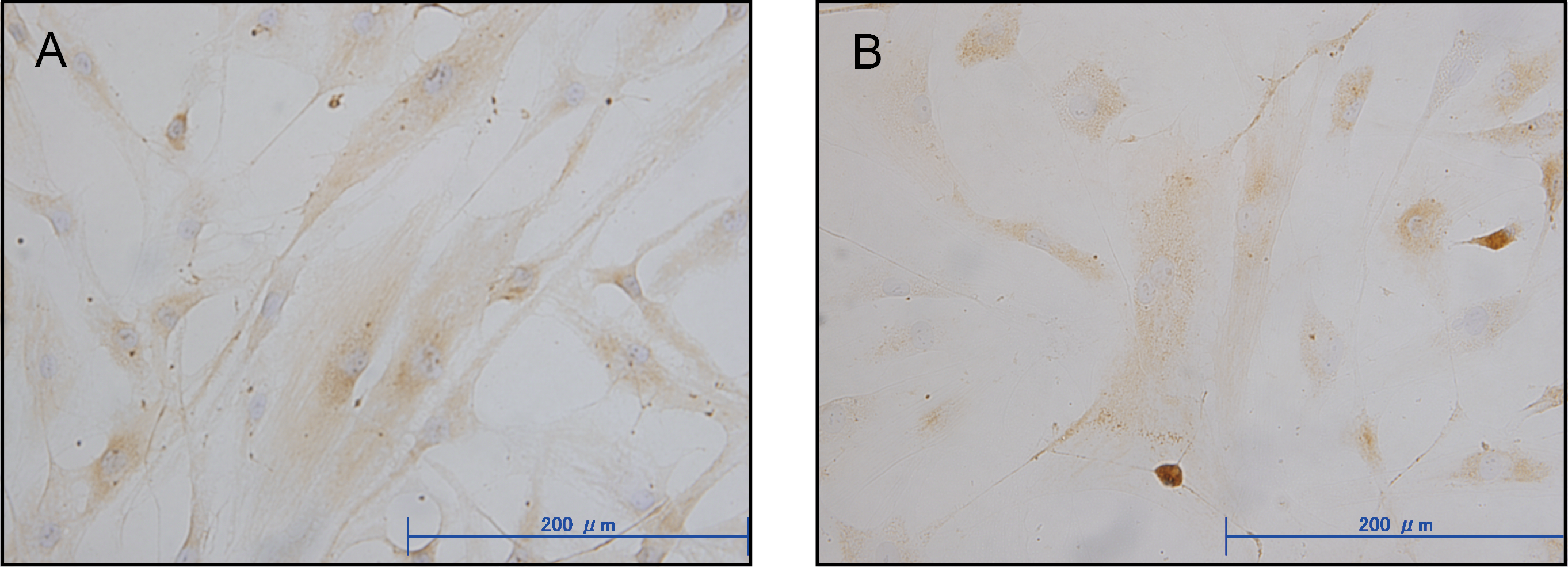

Supplement: S1 Fig — HA accumulation in RSFs after treatment with or without HAoligos for one hour was visualized by diaminobenzidine (DAB) staining using a biotinylated HA binding protein. Compared to results of longer incubation (three hours), slighter displacement of HMW-HA was observed after incubation with HAoligos for one hour. A, Control medium. B, 250 mg/ml HAoligos. Original magnification × 400, scale bar: 200 μm. (TIF) [file pone.0161875.s001.tif]

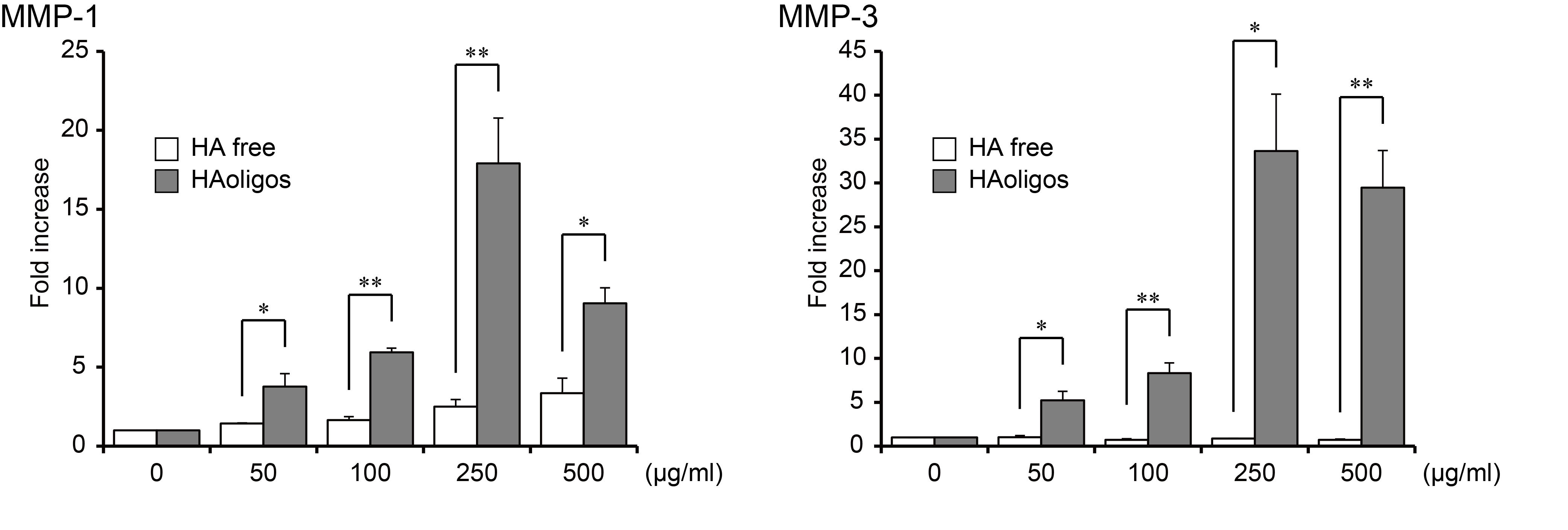

Supplement: S2 Fig — Hyaluronan (HA)-free solution was prepared in the same manner as the preparation of hyaluronan oligosaccharides, but without HMW-HA, and dialyzed with PBS in the final dialysis. HAoligos in PBS were also prepared using conventional methods and dialyzed with PBS in the final dialysis. The concentration of HAoligos in PBS was calculated using the weight after lyophilization. RSFs were treated with the same amount of HA-free solution as the amount of HAoligos in PBS. HAoligos in PBS significantly affected MMP mRNA expression, whereas the HA-free solution did not. *p<0.05 and **p<0.01 vs. HAoligos in PBS. (TIF) [file pone.0161875.s002.tif]

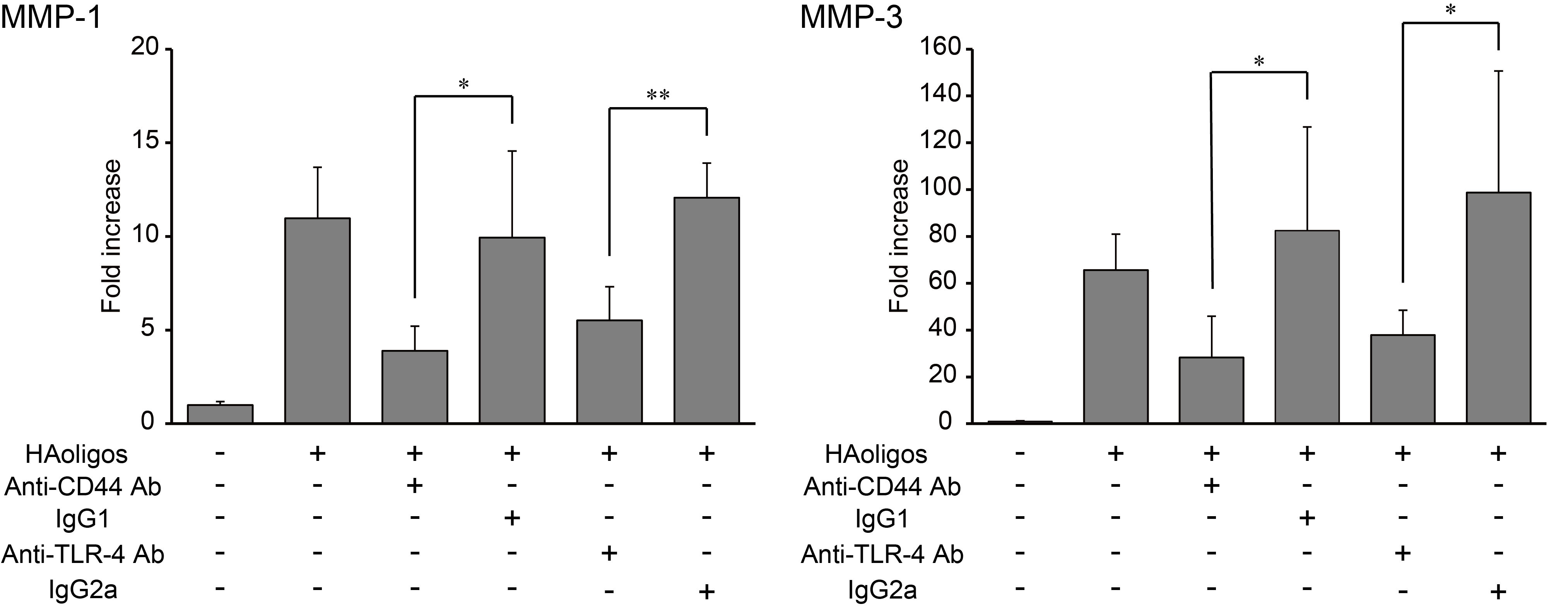

Supplement: S3 Fig — RSFs were pre-treated for one hour with or without antibodies (5 μg/ml), and followed by treatment with HAoligos (250 μg/ml) for 24 hours. MMP mRNA expression induced by HAoligos significantly decreased when RSFs were pre-treated with CD44 or TLR-4 neutralizing antibodies. In contrast, isotype-matched control IgG of anti-CD44 or -TLR4 antibodies did not reduce HAoligo-induced MMP mRNA expression. IgG1: isotype-matched control IgG of anti-CD44 antibody (Ancell). IgG2a: isotype-matched control IgG of anti-CD44 TLR4 antibody (Abcam). *p<0.05 and **p<0.01 vs. control IgG. (TIF) [file pone.0161875.s003.tif]

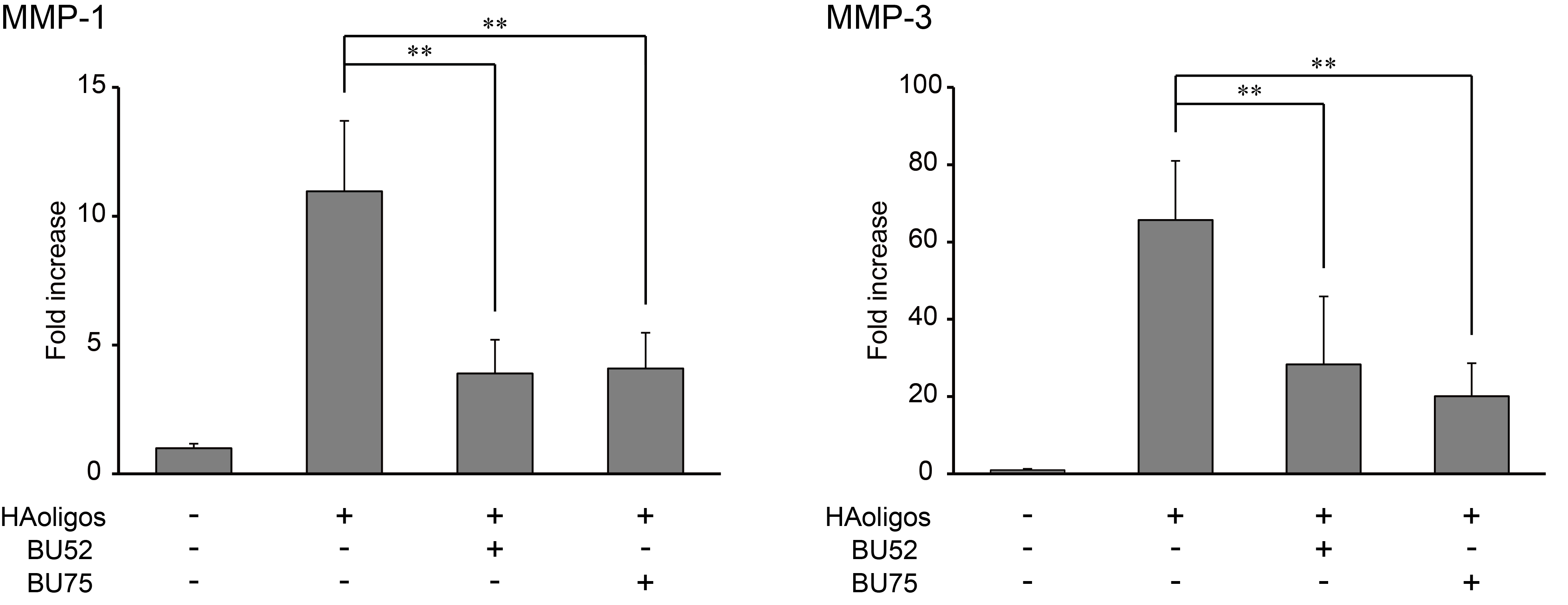

Supplement: S4 Fig — RSFs were pre-treated for one hour with or without antibodies (5 μg/ml), and followed by treatment with HAoligos (250 μg/ml) for 24 hours. The suppressive effect of the BU52 antibody on MMP mRNA expression induced by HAoligos was comparable to that of the BU75 antibody (Ancell). **p<0.01 vs HAoligos. (TIF) [file pone.0161875.s004.tif]

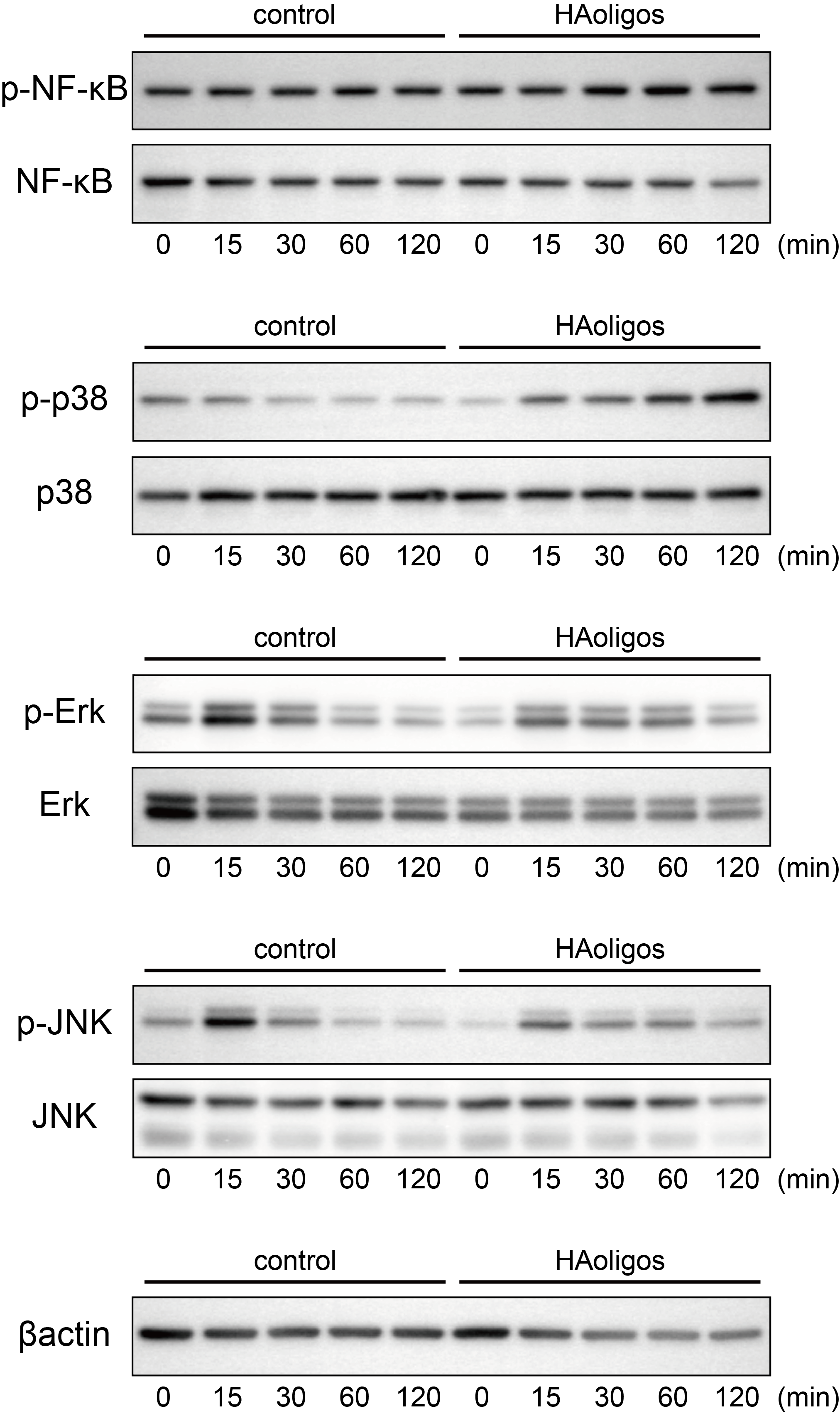

Supplement: S5 Fig — Total protein was extracted from RSFs treated for 0–120 minutes with or without HAoligos (250 μg/ml). Phosphate buffered saline was used as the control. Levels of phospho-NF-κB, NF-κB, phospho-p38 MAPK, p38 MAPK, phospho-Erk, Erk, phospho- JNK, and JNK were evaluated by immunoblot analysis. HAoligos enhanced the phosphorylation of NF-κB and p38 MAPK, while JNK and Erk phosphorylation levels were equivalent to control samples. (TIF) [file pone.0161875.s005.tif]
